# Effectiveness of different methods for delivering tailored nutrition education to low income, ethnically diverse adults

**DOI:** 10.1186/1479-5868-6-24

**Published:** 2009-05-05

**Authors:** Kim M Gans, Patricia M Risica, Leslie O Strolla, Leanne Fournier, Usree Kirtania, David Upegui, Julie Zhao, Tiffiney George, Suddhasatta Acharyya

**Affiliations:** 1 Brown University Institute for Community Health Promotion, Box G S121-8, Providence, RI 02912, USA; 2 Novartis Pharmaceutical Corporation, 1410 Sun Valley Way, Florham Park, NJ 07932, USA

## Abstract

**Background:**

Computer-tailored written nutrition interventions have been shown to be more effective than non-tailored materials in changing diet, but continued research is needed. Your Healthy Life/Su Vida Saludable (YHL-SVS) was an intervention study with low income, ethnically diverse, English and Spanish-speaking participants to determine which methods of delivering tailored written nutrition materials were most effective in lowering fat and increasing fruit and vegetable (F&V) intake.

**Methods:**

YHL-SVS was a randomized controlled trial with four experimental conditions: 1) Nontailored (NT) comparison group; 2) Single Tailored (ST) packet; 3) Multiple Tailored (MT) packet mailed in four installments; 4) Multiple Re-Tailored (MRT) MT packets re-tailored between mailings via brief phone surveys. A baseline telephone survey collected information for tailoring as well as evaluation. Follow-up evaluation surveys were collected 4- and 7-months later. Primary outcomes included F&V intake and fat related behaviors. Descriptive statistics, paired t-test and ANOVA were used to examine the effectiveness of different methods of delivering tailored nutrition information.

**Results:**

Both the ST and MT groups reported significantly higher F&V intake at 4-months than the NT and MRT groups. At 7 months, only the MT group still had significantly higher F&V intake compared to the NT group. For changes in fat-related behaviors, both the MT and MRT groups showed more change than NT at 4 months, but at 7 months, while these differences persisted, they were no longer statistically significant. There was a significant interaction of experimental group by education for change in F&V intake (P = .0085) with the lowest educational group demonstrating the most change.

**Conclusion:**

In this study, tailored interventions were more effective than non-tailored interventions in improving the short-term dietary behaviors of low income, ethnically diverse participants. Delivery of information in multiple smaller doses over time appeared to improve effectiveness. Future studies should determine which variables are mediators of dietary change and whether these differ by participant demographics. Moreover, future research should differentiate the effects of tailoring vs. cultural adaptation in ethnically diverse populations and study the dissemination of tailored interventions into community-based settings.

**Trial registration:**

Current Controlled Trials # NCT00301691.

## Introduction

A poor diet including inadequate F&V consumption and high fat consumption has been linked to overweight and obesity as well as several chronic diseases [1]. The average American does not meet the national guidelines for dietary intake [1-3]. Low-income, less educated and ethnic minority individuals may be even further from national goals [4-8]. Few self-help nutrition interventions have been developed for lower income or multi-ethnic audiences who often have lower literacy levels [9] and may lack knowledge and information about how to modify their diets [10].

Experts in developing materials for lower literate audiences recommend that an important way to improve communication and comprehension is to tailor educational materials, which involves personalizing the message to meet an individual participant's learning needs [11-14]. While tailoring has long been part of face-to-face counseling approaches, traditionally, it has been cost-prohibitive as a public health strategy. However, automated methods have made it feasible and inexpensive to produce nutrition education messages that are tailored for individuals based on their needs and characteristics. Studies have found that tailored nutrition interventions are effective in changing diet, but continued research is needed [11-16].

Most of the published studies with computer-tailored nutrition education have predominately involved Non-Hispanic white populations with higher education, literacy and income status. More remains to be learned about using tailored nutrition interventions to improve dietary behaviors with low income audiences from various ethnic minority groups [17,18]. Researchers should develop and test tailored programs that contain multiple versions geared to differences in demographics, language and other characteristics of specific populations [19]. In addition, information is needed on the most cost-effective modes of delivering tailored nutrition education programs to these populations [11,15,16,20-22].

Your Healthy Life/Su Vida Saludable (YHL/SVS) was a National Cancer Institute (NCI)-funded nutrition education intervention that sought to develop culturally- and linguistically-appropriate (English and Spanish) individually tailored nutrition education interventions, and to test these interventions as part of a randomized controlled trial with primarily low income, ethnically diverse adults to determine which methods of delivering tailored written nutrition materials were most effective and cost-effective in lowering fat and increasing F&V intake. The target audience was low-income Spanish or English speaking adults not already eating a healthy diet. This paper will present the final results of the intervention in terms of dietary change. Cost-effectiveness will be reported in a future paper.

## Methods

### Recruitment and Evaluation

This research was approved by the Brown University Institutional Review Board for Human Subjects. Participants were recruited and enrolled on a rolling basis. The majority of participant recruitment was conducted in the waiting rooms of nine public health clinics via face-to-face methods by research assistants. Additional recruitment took place in community centers, at public events, via a Spanish language radio show and through community partnerships. In addition, flyers and posters with a toll-free phone number were distributed throughout our target community. Interested individuals completed a registration form and informed consent either with the research assistant or by telephone. If found to be eligible, participants were later called to complete the baseline telephone survey. To be eligible, participants had to be at least 18 years old, able to read basic Spanish or English, not be pregnant, have no medical condition that precluded dietary changes, not already have a healthy diet (discussed below in Measures), have no significant hearing or visual impairments, have access to a VCR or DVD player and not live with another study participant.

Baseline surveys began in April 2003 and continued through October 2004. The baseline survey duration averaged 44 minutes in Spanish and 37 minutes in English. Participants received a $10 gift card for completing the baseline survey. A 20% random sample of baseline participants was also asked to complete a food frequency questionnaire (see below). These participants received an additional $10 incentive.

Upon completion of the baseline survey, study participants were randomized into one of four study groups: 1). Nontailored (NT) comparison group received a single mailing of non-tailored nutrition brochures purchased from national health promotion agencies that contained approximately 60 pages of nutrition messages related to lower fat and increased F&V; 2). Single Tailored (ST) received a single tailored educational packet; 3). Multiple Tailored (MT) received tailored information similar to the ST group, except that it was mailed in four installments over 12 weeks; 4). Multiple Re-tailored (MRT) also received tailored information in four installments, but the information in the three later mailings was re-tailored according to feedback collected via brief (average 7–8 minutes) telephone surveys prior to each of these mailings.

Follow-up evaluation surveys were collected by telephone 4- and 7-months after the start of the intervention by evaluation staff blinded to the participants' experimental condition. Follow-up surveys began in July 2003 and were completed in May 2005. The 4-and 7-month surveys averaged 32 and 30 minutes respectively, but were 5–7 minutes longer in Spanish than in English. Participants received a $10 gift card for completing each follow-up survey. To help increase response rates, this incentive was raised to $20 in 2004. In addition, in March of 2004, a telephone calling card incentive worth $5 was also added to each retailoring survey for MRT participants to increase response rates. All surveys were monitored for quality control purposes.

### Measures (surveys available upon request)

To measure F&V intake, the 7-item NCI Fruit and Vegetable screener assessment tool [23] was administered at all time points. This screener asks about the frequency of F&V intake as well as specific frequencies of juices, salad and potatoes. We adapted the paper and pencil form for telephone administration.

An adapted version of the Food Habits Questionnaire (FHQ) originally developed by Kristal and colleagues [24,25] was used to measure changes in fat-related dietary behaviors. This instrument assesses dietary behaviors that contribute to high-fat intake instead of focusing on foods and nutrients consumed [24-27]. We adapted the FHQ for use in YHL by adding questions about dietary behaviors related to bacon, ground meat, hotdogs and dining out that appeared to be important to overall fat intake based on our formative research [28] (discussed below). We also culturally targeted the instrument for the Latino audience by translating it into Spanish, adding questions about preparation of rice and beans, and adding examples of culturally relevant foods to selected questions. For example, the items chicharrones (pork rinds) and platanos fritos (fried green plantain) were added to the question about salty snacks. These adaptations were guided by the formative research as well as by our Latino staff.

Each FHQ behavioral item was introduced by a question asking whether or not the participant ate a particular food. Response categories were *yes/no*. If the participant indicated that they ate a particular food, they were asked the related behavioral questions. If they did not eat the food, they skipped to the next introductory question. Five response categories for the behavioral questions were: 0 = almost always, 1 = often, 2 = sometimes, 3 = rarely, and 4 = never. Ten items were reverse scored prior to analysis. The FHQ fat summary score was calculated by taking the mean of all behavioral FHQ questions. A lower FHQ score indicates a higher prevalence of fat-lowering behaviors and thus reflects a lower fat intake. Participants were deemed ineligible if their FHQ responses determined that they had fewer than four FHQ food behavior categories that had an opportunity to be improved (i.e. had responses of sometimes, rarely or never).

In order to calibrate the adapted FHQ, we collected nutrient data using a food frequency questionnaire (FFQ) developed by the Fred Hutchinson Cancer Research Center [26,29] from 20% of the participants chosen randomly at baseline. After completing an adequate number of FFQ surveys for calibration purposes (n = 188), administration of the FFQ was stopped to decrease respondent burden. The correlation of the baseline FHQ score to dietary fat in grams was 0.49 for (p < .0001) and 0.46 for percent calories from fat (p < .0001), which indicated that the FHQ score does reflect dietary fat intake.

The baseline survey also included questions regarding demographics, acculturation when appropriate, self-reported height and weight and medical history. In addition to evaluation questions, baseline surveys included questions for tailoring purposes (see below) and the follow-up surveys included process evaluation questions.

### Intervention

The development of the YHL/SVS intervention was informed by formative research to ascertain the determinants of dietary behavior in the target populations and to develop specific intervention messages that could impact on these determinants to change dietary behavior in ways relevant to the target audience. The formative research and the development of the intervention are described in detail elsewhere [28]. Briefly, qualitative data gathered from six focus groups and 20 individual interviews were used to determine and explain the factors that influence food-related choices and behaviors in our target populations. This information was supplemented and expanded with data from a quantitative telephone survey (n = 334) to determine the frequency of these factors in a more representative cross-section of the target population. These results guided the design and development of the intervention and evaluation materials, which were then confirmed and pre-tested with an additional five focus groups and an individual interview. A total of 474 participants took part in the formative research [28].

The YHL-SVS tailored intervention was based on theoretical constructs from the Transtheoretical Model [30,31] and Social Cognitive Theory [32] to impact on determinants that included perceived pros (motivators) and cons (barriers) for dietary change, stage of change, social support, and situational self-efficacy. The library of written materials included 21 nutrition topics with 127 different pages of content. The specific subset of pages and messages received by an individual depended on their answers to the baseline survey and retailoring surveys (MRT only).

**Figure 1:**
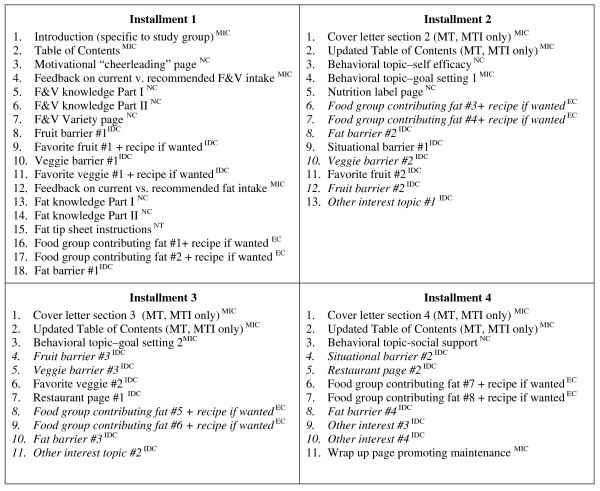
**Tailored content included in each installment of YHL-SVS intervention materials**.

Tailoring was done in two ways: macro-tailoring, where an entire page was chosen for a participant; and micro-tailoring, where the tailored message or graphic was merged into a document template. Micro-tailoring was also used to give participants individualized feedback on their F&V intake as well as specific dietary behaviors they could change to reduce fat intake. Macro-tailored pages were chosen by computer algorithms, by participant choice or by a combination of both. Most pages were personalized with the participant's name. Figure 1 describes the pages in each installment of the tailored intervention packet and how the information was tailored.

For those participants in the MRT group, the retailoring telephone surveys re-asked baseline tailoring questions about situational self-efficacy and barriers to fruit, vegetable and fat consumption. Participants also had the opportunity to reprioritize pages related to fat behaviors, restaurants and special interest topics that they were most interested in. Figure 1 identifies the specific pages that were retailored. If participants could not be reached to complete these surveys, their next set of materials was tailored based on their answers to the baseline survey and/or previous retailoring survey. Installments of materials were mailed in weeks 1, 5, 9, and13 with retailoring by phone in weeks 3, 7, and 11.

The tailored information was mailed in a colorful 3-ring binder which also included a refrigerator magnet shopping list with the project logo and toll-free number. The binder also included a brief (approx. 10 minute) motivational and instructional DVD/video targeted to the participant's study group, ethnicity (Hispanic or Non-Hispanic) and language (English or Spanish). All versions of the DVD/video presented testimonials by target audience members that addressed the five motivators for eating healthier that were found to be most prevalent in formative research: look great/lose weight; feel good about yourself; be healthy; for family to eat better; and feel better.

There were three versions of all materials and videos: English, Spanish and English for Hispanics. Translation of Spanish materials was done by a professional translation firm with confirmation by Hispanic staff members. The materials for Hispanics substituted pictures, graphics, food issues and choices that were culturally relevant to Hispanics [28]. The written materials were tested for readability and the English materials were found to be at 6th grade level or lower, as measured by the Flesch-Kincaid Grade level score. Spanish materials were tested for readability via focus groups with members of the target population who read and discussed the pages as well as by Cloze testing [33] to assess understanding.

## Statistical methods

All statistical analyses were performed with SAS software version 8.2 (SAS Institute, Cary, NC). Descriptive summary statistics, (such as means and proportions, along with their respective standard deviations) were reported for the overall sample and for individual experimental and ethnic (Hispanic vs. Non-Hispanic) groups. Since no significant differences were observed when comparing demographic traits across study groups, adjustment for demographic characteristics in models evaluating group differences was not deemed necessary.

All FHQ scores more than 3 standard deviations from the mean were considered outliers, and excluded from the calculations, which omitted four participants. Paired t-tests were performed to test for differences from baseline at 4 and 7 months, with respect to F&V servings (fruit without juice; vegetables without French fries) and FHQ scores. Analysis of variance models were constructed with F&V servings and FHQ scores at 4 or 7 months follow up as the dependent variable, and experimental group as the main explanatory variable. The models were adjusted for the baseline value of the dependent variable. Analyses of the intervention effect on mean differences in F&V intake and FHQ score (dietary fat) were also performed for each follow-up time point on the raw data, and also on an intent-to-treat basis. Two methods were employed for imputing missing follow-up values, viz. LOCF (Last Observation Carried Forward) and 'mean substitution', whereby missing values in the experimental group were substituted by the corresponding means calculated for the control group. Inferences from the two methods were similar, thus, results based on LOCF alone are reported in the interest of space.

The influence of education, income level, and ethnicity on the outcome of the intervention on dietary practices was also assessed. Of the three, the interaction of education (categorized into two levels viz. 'less than high school', and 'high school or more') with experimental group turned out to be significant, so changes at each level of education were explored separately.

## Results

**Figure 2:**
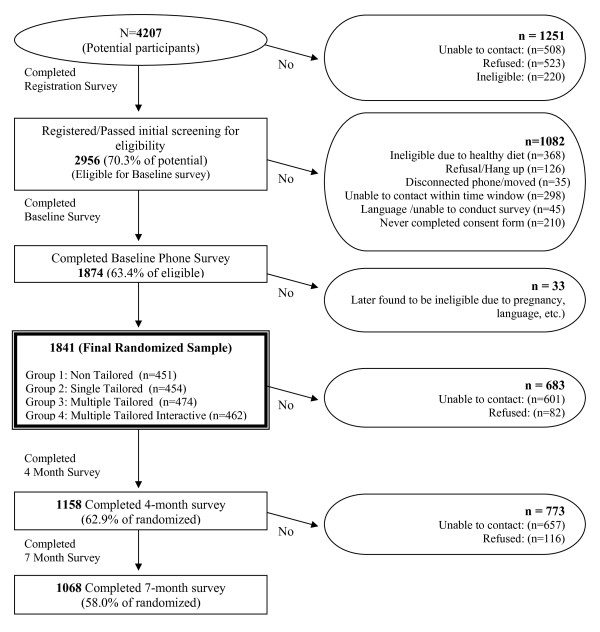
**YHL-SVS sample size flow chart**.

Figure 2 shows the flow chart of study participation. The final baseline sample included 1841 participants. Table 1 describes the demographics of the final sample by ethnicity. Overall, 55% of participants self-identified as Hispanic. Although the intervention was not designed to specifically target Black/African-Americans, 12.5% of participants self-identified as such. Only 9% of Hispanic participants reported being born in the United States with the rest born in a diverse group of countries including the Dominican Republic (32%), Puerto Rico (19%); Colombia (12%); Guatemala (11%); Mexico (5%); and Bolivia (4%) (data not shown). The majority of participants were female, low-income (household income less than $20,000) and most had a high school education or less. Two thirds of study participants reported having children in their household. There were no differences between experimental groups at baseline for any of the demographic characteristics (data not shown).

**Table 1:** Baseline demographics by ethnicity (n = 1841)

**VARIABLE**	**HISPANIC**	**NON-HISPANIC**	**P-VALUE**	**TOTAL**
**FEMALE**	86.3%	83.6%	NS	85.1%

				

**EMPLOYED**	49.8%	53.1%	NS	51.3%

				

**EDUCATION**				

< High School	42.1%	24.0%	< .0001	33.9%

High School Graduate	29.1%	32.3%		30.5%

> High School	28.8%	43.8%		35.6%

				

**MARRIED**				

Currently Married	43.4%	30.4%	< .0001	37.5%

Never Married	30.6%	36.9%		33.5%

Previously Married	26.0%	32.8%		29.1%

				

**INCOME**				

<= 20 K	62.0%	49.6%	< .0001	56.4%

21 – 40 K	22.2%	25.2%		23.6%

41 K +	3.8%	17.3%		9.9%

				

**AGE GROUP**				

18–34	41.9%	30.3%	< .0001	36.7%

35–51	41.6%	44.6%		43.0%

52+	16.5%	25.1%		20.4%

				

**MEAN AGE**	38.7 (SD 12.2)	42.6 (SD 13.6)	< .0001	40.4 (SD 12.9)

				

**CHILDREN IN HOUSEHOLD**	73.7%	56.7%	< .0001	66.0%

				

**RACE**				

American Indian	3.6%	4.9%	< .0001	4.2%

Asian	0%	1.6%		0.7%

Black/African American	3.7%	23.2%		12.5%

Native HI/Pacific Is.	0.7%	0.1%		0.4%

White/Caucasian	16.0%	59.2%		35.6%

More than one race	60.6%	10.3%		37.9%

Unknown	15.4%	0.7%		8.7%

**TOTAL**	**1007**	**834**		**1841**

Follow-up response rates on the final sample of 1841 were 63% for the 4 month survey and 58% for the 7 month survey. The main reason that participants did not complete a follow-up survey was because they could not be reached despite varied approaches including up to 19 call attempts on different days and times over a 28 day period, online searches, help letters, calling contact people provided at registration, and community networking.

After data collection was complete, analyses were performed to determine if follow-up rates differed by experimental condition and/or demographics. No significant differences in follow-up rates between experimental groups were identified (p = 0.76). However, completers of follow-up surveys were more likely to be female, live alone, be better educated, married, older, and prefer their study materials in Spanish than participants completely lost-to-follow-up (data not shown).

### Process Evaluation

All the tailored intervention and comparison groups were receptive to the materials they received. When asked at follow-up, 77% of participants in the tailored intervention groups reported having read most or all of the tailored pages; 14% reported reading some pages and 85% reported that they were still using the materials at 4-months with 86% still using them at 7-months. Over 70% of participants reported watching at least some of the motivational video, with almost half reporting that they watched it all. Over 96% thought the written materials were very or somewhat helpful and over 97% would recommend the program to others. Over 70% of this group reported sharing their tailored materials with others. Completion rates for retailoring surveys were relatively high, but decreased with each subsequent survey (86.2%, 76.4% and 70.1%, respectively).

NT comparison group participants were also pleased with their materials with 70% reporting that they had read all or most and 21% reported reading some of the materials. Over 90% found the materials very or somewhat helpful and 59% reported they were still using them at 4 months with 69% still using them at 7 months. More than 96% of NT participants would recommend the program to others and 64% reported sharing the materials with others.

### Dietary change

Overall, all experimental groups demonstrated significant changes in FHQ score (fat) as well as F&V intake from baseline to 4 and 7 month follow-up (Table 2). Both the ST and MT groups reported significantly higher F&V intake at 4-months than the NT and MRT groups. For the ST and NT interventions there was some recidivism in F&V intake between 4 and 7 months, but the MT group maintained their F&V increases and the MRT group increased intake between 4 and 7 months. At 7 months, the MT group still had significantly higher F&V intake compared to the NT group (p < .02), but no other group differences were significant.

**Table 2:** Fruit and vegetable and Food Habit Questionnaire (FHQ) score changes at 4 and 7 months by experimental group using intent-to treat analyses

	NT Mean (STD)	ST Mean (STD)	MT Mean (STD)	MTI Mean (STD)
4 Month Change in Fruit and Vegetable Servings	0.42 (2.51)^b, c^	0.92 (2.92)^a, d^	0.72 (2.55)^a, d^	0.36 (2.58)^b, c^

P different from 0	.0004	<.001	<.001	<.001

				

7 Month Change in Fruit and Vegetable Servings	0.24 (2.52)^c^	0.58 (2.69)	0.68 (2.63)^a^	0.49 (2.58)
P different from 0	.05	<.001	<.001	<.001

				

4 Month Change in Dietary Fat (FHQ Score)	-0.27 (0.44)^c, d^	-0.28 (0.47)^c^	-0.31 (0.50)^a, b^	-0.32 (0.51)^a^

P different from 0	<.001	<.001	<.001	<.001

				

7 Month Change in Dietary Fat (FHQ Score)	-0.27 (0.48)	-0.29 (0.49)	-0.31 (0.48)	-0.32 (0.51)

P different from 0	<.001	<.001	<.001	<.001

a = significantly different from NT; b = significantly different from ST;

c = significantly different from MT; d = significantly different from MTI

4 month Fruit and Veg: ST v NT p = .01, MT v. NT p = .05, ST v. MTI p = .01, MT v. MTI p = .01

7 month Fruit and Veg: MT v. NT p = .02

4 month FHQ: MT vs. NT p = .01, MTI vs. NT p = .02, MT vs. ST p = .04

For changes in the fat-related behaviors measured by the FHQ, both the MT and MRT groups showed more change than NT (p = .01 and .02 respectively) at 4 months. All groups maintained their decreases in fat between 4 and 7 months, but differences between groups were no longer statistically significant at 7 months. NT and ST did not differ in FHQ score at either follow up. FHQ score for MT was significantly lower (p = .04) at 4 months compared to ST, but did not differ at 7 months. Neither ST nor MT were different from MRT at either time point.

Further data analyses showed that group differences in dietary change were statistically significant for the interaction of group by education for change in F&V intake at 7 months follow-up (p = .0085) only (data not shown). Data comparing the NT intervention to the combined tailored interventions (ST, MT, MRT) by demographic characteristics indicated that the tailored interventions were more effective for participants with less than a high school education than those with a high school education or more. The less educated group demonstrated a 1.65 serving greater increase at 7 months (p < .0001) (data not shown).

## Discussion

Overall, this study demonstrates that it is possible to develop tailored nutrition education materials at low literacy levels in English and Spanish that are read and well-liked by low income participants and effective in achieving dietary change. Moreover, we were able to recruit a large number of low income, ethnically diverse participants and achieved high rates of intervention delivery. Tailoring was shown to be more effective than non-tailored nutrition education materials in this predominantly low income, ethnically diverse population, and was more effective in lower educated than higher educated study participants. This is the only published study to date that has conducted tailoring simultaneously in more than one language and only the second study demonstrating that tailoring in Spanish is more effective than non-tailored materials in changing dietary habits with a Hispanic audience [34,35]. Other studies have shown that tailored nutrition materials are more effective than non-tailored interventions for African Americans [36,37] and for low income populations [37,38] but others have not [19].

Resnicow et al recommends assessing individual differences as potential moderators of tailored health interventions [39]. Even though YHL-SVS was not powered to look at subgroup differences, this study did find that the tailored intervention was even more effective in increasing F&V intake for lower educated than higher educated individuals. This may be partly due to the low literate nature of the educational materials, but it also may be that tailored materials are particularly more effective for lower educated individuals because they are more personally relevant [40-42]. Brug et al found no difference in the impact of computer-tailored feedback on fat intake between high and low educational groups, but they did find that lower educated respondents were more positive about how interesting and personally relevant the tailored letters were [42]. The relative effectiveness of tailored nutrition materials on population subgroups should be studied further in appropriately powered studies.

Future studies should also look at psychosocial mediators of change and whether mediators differ by demographic characteristics. Moreover, YHL-SVS involved both cultural "targeting" of materials as well as individual tailoring on behavioral and psychosocial constructs. Future studies should differentiate the effects of each. Kreuter et al found that tailoring on both behavioral and cultural constructs (religiosity, collectivism, racial pride, and time orientation) was more effective than behaviorally tailored or culturally tailored materials alone in increasing F&V consumption in a low income sample of African American women [37]. More research is needed on defining appropriate cultural constructs for other ethnic minority groups (including Hispanics) and testing these in nutrition interventions with and without behavioral construct tailoring.

The results of the YHL-SVS randomized trial indicate that all of the interventions, including the NT intervention resulted in significant dietary changes. This is likely somewhat due to social desirability bias (discussed further below), but is also likely because these low income populations had not been exposed to much nutrition education prior to this study.

Overall, tailored materials in general, and multiple tailored mailings in particular, were more effective in increasing F&V and reducing fat intake than non-tailored materials. For increasing F&V intake the ST and MT interventions were superior to the NT and MRT interventions at 4 months, but at 7 months, only the MT group was superior to the NT group. In contrast, for fat intake, MT and MRT interventions both had significantly higher decreases than the NT group and MT had more change than ST at 4 months. Differences in fat intake between groups persisted at 7 months but were no longer statistically significant due to losses in power because of a smaller sample size. The different effects of the various tailoring methods on F&V compared to fat intake at 4 months may be because lowering fat intake is a more complex behavior than increasing F&V intake. Reducing fat requires making multiple changes in food choices as well as changes in food preparation. Thus, multiple smaller doses of information (MT) may be easier to "digest" and incorporate into one's lifestyle than one large dose of information (ST) for a complex behavior like reducing dietary fat. In contrast, increasing F&V intake is a less complex behavior; thus, a single large dose of tailored information was effective for changing this behavior, at least in the short term.

Overall, the MRT group was not better in achieving dietary change than MT, thus, implementing retailoring in the manner in which it was done in YHL/SVS is not recommended as it uses more resources. It is unclear if other types of retailoring such as reassessing diet might have fared better. We did not reassess diet because of concerns about respondent burden with our lengthy dietary assessment tools. Even the three brief retailoring calls appear to have been burdensome and more contact than many participants found helpful. Further research should elucidate if different methods of retailoring such as by mail or computer are more cost effective and on which variables researchers should provide retailoring. Whether tailored interventions (and retailoring) need to include a combination of participant choice and computer choice like in the YHL/SVS study, or whether one or the other is sufficient or better for changing dietary behavior should also be explored.

Two other studies have looked at multiple vs. single tailoring and retailoring for nutrition education. Brug et al studied the impact of the additional effects of iterative feedback ("retailoring") on changes in intake of fat and F&V [43]. Respondents in the experimental group received letters tailored to their dietary intake, intentions, attitudes, self-efficacy and self-rated behavior. After the first tailored letter, half of the experimental group received additional iterative feedback tailored to changes in behavior and intentions. The control group received a single general nutrition information letter. The tailored intervention had a significantly greater impact on fat reduction and F&V intake than did general information and iterative tailored feedback had an additional impact on fat intake, but not on F&V intake [43]. However, this study could not discern whether the additional dietary change was due to the retailoring or the additional contact with the participant.

In another study, Heimendinger et al gave callers to the NCI's Cancer Information Service a telephone interview including a brief educational message and then randomized participants to one of four groups: single untailored (SU) group receiving one untailored set of materials; single tailored (ST) group receiving one tailored booklet; multiple tailored (MT) group receiving the tailored booklet plus three additional tailored mailings; and multiple retailored (MRT) group receiving the MT intervention with retailoring based on new information obtained at 5 months follow-up [44]. MT and MRT were both more effective at increasing F&V consumption than were SU materials, but the MRT materials were not more effective than MT [44]. These results are similar to YHL/SVS; however the Heimindinger study demonstrated the effectiveness of both higher dose and multiple mailings combined, while YHL/SVS was able to clearly demonstrate the effectiveness of multiple mailings alone because dose was constant between groups.

Before discussing the study implications, it is important to mention several study limitations. The assessment tool that measured dietary change (FHQ) did not include a quantitative measure of fat intake (i.e. percent calories from fat); however, this tool has been used in other studies and was calibrated in the current study against a quantitative measure and found to reflect dietary fat intake. In addition, recent studies have shown that the NCI F&V screener may overestimate F&V intake [45,46], but this would not have affected differences in F&V intake by group. Another limitation is that there was no measure of social desirability bias, which may have accounted for some of the observed effects. However, as all participants received the same amount of information on fat and F&V and there were no differences in survey content or staff contact (with the exception of the MRT group, which did not show increased dietary change compared to the other groups) it is likely that such bias would have occurred in all experimental groups.

In addition, because all the tailored interventions included an untailored video as well as tailored written information, the effect of the video on dietary change cannot be differentiated from the effect of the written information. However, it is unlikely that the brief video, which contained motivational testimonials and instructions on how to use the tailored binder but no dietary change content, would have much effect compared to the multiple pages of tailored nutrition information. Furthermore, differential effects between tailored groups could not be confounded by the video.

The study did not include a long term measure of dietary change (i.e. 12 months or longer). YHL-SVS originally did include a 12 month follow-up measure, but this follow-up was shortened to 7 months when funding was cut prior to the start of the study. Future studies should measure whether such tailored interventions maintain dietary changes over a longer timeframe.

Follow-up rates for evaluation in YHL-SVS were disappointing even though many attempts and various methods were used to contact participants. To control for possible bias associated with differences among respondents versus non-responders, intention-to-treat analyses were used. Attrition may have been due to several factors. In low-income populations there is a great deal of transience as well as disruption of telephone service. Furthermore, the majority of study recruitment was conducted via active face-to-face methods by a bilingual research assistant who was very friendly and engaging. Thus, some individuals that were not completely committed to the study may have been convinced to join. Other studies have shown that active recruitment methods yield higher study enrollment rates, but lower retention rates than passive recruitment methods [47]. Moreover, because the intervention and evaluation surveys were done by mail and telephone with no face-to-face contact after recruitment, there was not an opportunity for research participants to develop a rapport or "personalismo" with study staff, which may be particularly important for retaining Hispanic participants. In addition, we heard from study participants that the baseline telephone survey was too long and this likely discouraged some participants from wanting to complete follow-up telephone surveys. Study incentives of only $10–20 may not have been high enough to encourage participants to complete the follow-up surveys. Further research is needed on retention of low income, ethnically diverse participants in nutrition intervention studies.

## Conclusions and future implications

Tailored interventions delivered in English and Spanish are more effective than non-tailored interventions in improving the short-term dietary behaviors of low income, ethnically diverse participants and delivering the information in multiple smaller doses over time appears to be the most effective method. Such interventions may be especially potent for lower educated individuals. Retailoring was not shown to be cost effective in this study.

Future studies should determine which questions/variables are most important for tailoring, retailoring and evaluation so that surveys can be as brief as possible to decrease respondent burden and increase study retention. In addition, future research should examine mechanisms that explain why tailoring is more effective and how these mechanisms may differ by population subgroups and for different dietary variables. Moreover, future research should differentiate the effects of cultural vs. behavioral construct tailoring in various ethnic minority groups. Additional channels for delivering nutrition tailored interventions such as video, computer and internet should be studied with low income, ethnically diverse populations. It is also very important to find ways to disseminate effective tailored nutrition interventions. The dissemination of YHL-SVS to community-based settings serving low income, ethnically diverse consumers is currently being studied by our research group in a translational research study funded by the Centers for Disease Control.

## Competing interests

The authors declare that they have no competing interests.

## Authors' contributions

KG was Principal Investigator of the study. She conceived of the study, participated in its design and coordination and took the lead in writing the manuscript. PR was Co-Principal Investigator of the study and helped to draft the manuscript, in particular the methods section. LS was Program Coordinator and helped to draft the manuscript, in particular the intervention section and the intervention tables. UK performed the statistical analysis and wrote most of the statistical analysis section and most of the tables. LF reviewed and edited the entire manuscript, and contributed to the discussion and intervention tables. DU was the data manager for the study. He provided data for the results section as well as the flow chart figures. JZ performed the interaction analysis and wrote this section of the methods and results. TG is an MPH student who conducted a literature review that contributed to the introduction and discussion sections. SA is a biostatistician who was previously employed by Brown University. He provided statistical expertise and assisted with the interpretation of the results and in writing the statistical methods section. All authors read and approved the final manuscript.

## References

[B1] U.S. Department of Health and Human Services, U.S. Department of Agriculture (2005). Nutrition and Your Health: Dietary Guidelines for Americans.

[B2] American Heart Association (2004). Heart Disease and Stroke Statistics – 2005 Update.

[B3] Popkin BM (2001). The nutrition transition and obesity in the developing world. J Nutr.

[B4] Patterson BH, Krebs-Smith SM, Subar AF (1997). Correction and revision of conclusions – dietary trends in the United States. N Engl J Med.

[B5] Kumanyika S (1996). Improving our diet – still a long way to go. N Engl J Med.

[B6] Patterson RE, Kristal AR, Coates RJ, Tylavsky FA, Ritenbaugh C, Van Horn L, Caggiula AW, Snetselaar L (1996). Low-fat diet practices of older women: prevalence and implications for dietary assessment. J Am Diet Assoc.

[B7] Bialostosky K, Wright JD, Kennedy-Stephenson J, McDowell M, Johnson CL (2002). Dietary intake of macronutrients, micronutrients, and other dietary constituents: United States 1988–94. Vital Health Stat 11.

[B8] U.S. Department of Health and Human Services (2000). Healthy People 2010: Understanding and Improving Health.

[B9] Winkleby MA, Howard-Pitney B, Albright CA, Bruce B, Kraemer HC, Fortmann SP (1997). Predicting achievement of a low-fat diet: a nutrition intervention for adults with low literacy skills. Preventive medicine.

[B10] Contento IR, Black GI, Bronner Y, Lytle L, Maloney SK, Olson CM, Swadener SS (1995). The effectiveness of nutrition education and implications for nutrition education policy, programs and research: A review of research. J Nutr Educ.

[B11] De Vries H, Brug J (1999). Computer-tailored interventions motivating people to adopt health promoting behaviours: introduction to a new approach. Patient Educ Couns.

[B12] Brug J, Oenema A, Campbell M (2003). Past, present, and future of computer-tailored nutrition education. Am J Clin Nutr.

[B13] Kroeze W, Werkman A, Brug J (2006). A systematic review of randomized trials on the effectiveness of computer-tailored education on physical activity and dietary behaviors. Annals of Behavioral Medicine.

[B14] Noar SM, Benac CN, Harris MS (2007). Does tailoring matter? Meta-analytic review of tailored print health behavior change interventions. Psychol Bull.

[B15] Brug J, Steenhuis I, van Assema P, Glanz K, De Vries H (1999). Computer-tailored nutrition education: differences between two interventions. Health Education Research.

[B16] Dijkstra A, De Vries H (1999). The development of computer-generated tailored interventions. Patient Educ Couns.

[B17] Skinner CS, Strecher VJ, Hospers H (1994). Physicians' recommendations for mammography: do tailored messages make a difference?. Am J Public Health.

[B18] Rimer BK, Glassman B (1998). Tailoring communications for primary care settings. Methods Inf Med.

[B19] Campbell MK, Carbone E, Honess-Morreale L, Heisler-Mackinnon J, Demissie S, Farrell D (2004). Randomized trial of a tailored nutrition education CD-ROM program for women receiving food assistance. Journal of Nutrition Education and Behavior.

[B20] Campbell MK, Bernhardt JM, Waldmiller M, Jackson B, Potenziani D, Weathers B, Demissie S (1999). Varying the message source in computer-tailored nutrition education. Patient Educ Couns.

[B21] Brug J, Campbell M, van Assema P (1999). The application and impact of computer-generated personalized nutrition education: a review of the literature. Patient Education and Counseling.

[B22] Miller SM, Bowen DJ, Campbell MK, Diefenbach MA, Gritz ER, Jacobsen PB, Stefanek M, Fang CY, Lazovich D, Sherman KA, Wang C (2004). Current research promises and challenges in behavioral oncology: report from the American Society of Preventive Oncology annual meeting, 2002. Cancer Epidemiol Biomarkers Prev.

[B23] Thompson FE, Byers T (1994). Dietary assessment resource manual. J Nutr.

[B24] Shannon J, Kristal AR, Curry SJ, Beresford SA (1997). Application of a behavioral approach to measuring dietary change: the fat- and fiber-related diet behavior questionnaire. Cancer Epidemiol Biomarkers Prev.

[B25] Kristal AR, White E, Shattuck AL, Curry S, Anderson GL, Fowler A, Urban N (1992). Long-term maintenance of a low-fat diet: durability of fat-related dietary habits in the Women's Health Trial. J Am Diet Assoc.

[B26] Kristal AR, Beresford SA, Lazovich D (1994). Assessing change in diet-intervention research. Am J Clin Nutr.

[B27] Kristal AR, Shattuck AL, Patterson RE (1999). Differences in fat-related dietary patterns between black, Hispanic and White women: results from the Women's Health Trial Feasibility Study in Minority Populations. Public Health Nutr.

[B28] Strolla LO, Gans KM, Risica PM (2006). Using qualitative and quantitative formative research to develop tailored nutrition intervention materials for a diverse low-income audience. Health Educ Res.

[B29] Kristal AR, Vizenor NC, Patterson RE, Neuhouser ML, Shattuck AL, McLerran D (2000). Precision and bias of food frequency-based measures of fruit and vegetable intakes. Cancer Epidemiol Biomarkers Prev.

[B30] Prochaska JO, DiClemente CC (1983). Stages and processes of self-change of smoking: toward an integrative model of change. Journal of consulting and clinical psychology.

[B31] Prochaska JO, DiClemente CC, Miller WR (1986). Toward a comprehensive model of change. Treating Addictive Behavior: Process of Change.

[B32] Bandura A (1986). Social foundations of thought and action: a social cognitive theory.

[B33] Taylor WL (1953). Cloze procedure: A new tool for measuring readability. Journalism Quarterly.

[B34] Elder JP, Ayala GX, Campbell NR, Slymen D, Lopez-Madurga ET, Engelberg M, Baquero B (2005). Interpersonal and print nutrition communication for a Spanish-dominant Latino population: Secretos de la Buena Vida. Health Psychol.

[B35] Elder JP, Ayala GX, Campbell NR, Arredondo EM, Slymen DJ, Baquero B, Zive M, Ganiats TG, Engelberg M (2006). Long-term effects of a communication intervention for Spanish-dominant Latinas. Am J Prev Med.

[B36] Di Noia J, Schinke SP, Prochaska JO, Contento IR (2006). Application of the transtheoretical model to fruit and vegetable consumption among economically disadvantaged African-American adolescents: preliminary findings. Am J Health Promot.

[B37] Kreuter MW, Sugg-Skinner C, Holt CL, Clark EM, Haire-Joshu D, Fu Q, Booker AC, Steger-May K, Bucholtz D (2005). Cultural tailoring for mammography and fruit and vegetable intake among low-income African-American women in urban public health centers. Prev Med.

[B38] Nitzke S, Kritsch K, Boeckner L, Greene G, Hoerr S, Horacek T, Kattelmann K, Lohse B, Oakland M, Phillips B, White A (2007). A Stage-tailored Multi-modal Intervention Increases Fruit and Vegetable Intakes of Low-income Young Adults. Am J Health Promot.

[B39] Resnicow K, Davis RE, Zhang G, Konkel J, Strecher VJ, Shaikh AR, Tolsma D, Calvi J, Alexander G, Anderson JP, Wiese C (2008). Tailoring a fruit and vegetable intervention on novel motivational constructs: results of a randomized study. Ann Behav Med.

[B40] Oenema A, Tan F, Brug J (2005). Short-term efficacy of a web-based computer-tailored nutrition intervention: main effects and mediators. Ann Behav Med.

[B41] Oenema A, Brug J, Lechner L (2001). Web-based tailored nutrition education: results of a randomized controlled trial. Health Education Research.

[B42] Brug J, van Assema P (2000). Differences in use and impact of computer-tailored dietary fat-feedback according to stage of change and education. Appetite.

[B43] Brug J, Glanz K, Van Assema P, Kok G, van Breukelen GJ (1998). The impact of computer-tailored feedback and iterative feedback on fat, fruit, and vegetable intake. Health Education & Behavior.

[B44] Heimendinger J, O'Neill C, Marcus AC, Wolfe P, Julesburg K, Morra M, Allen A, Davis S, Mowad L, Perocchia RS, Ward JD, Strecher V, Warnecke R, Nowak M, Graf I, Fairclough D, Bryant L, Lipkus I (2005). Multiple tailored messages are effective in increasing fruit and vegetable consumption among callers to the Cancer Information Service. Journal of health communication.

[B45] Greene GW, Resnicow K, Thompson FE, Peterson KE, Hurley TG, Hebert JR, Toobert DJ, Williams GC, Elliot DL, Goldman Sher T, Domas A, Midthune D, Stacewicz-Sapuntzakis M, Yaroch AL, Nebeling L (2008). Correspondence of the NCI Fruit and Vegetable Screener to repeat 24-H recalls and serum carotenoids in behavioral intervention trials. J Nutr.

[B46] Peterson KE, Hebert JR, Hurley TG, Resnicow K, Thompson FE, Greene GW, Shaikh AR, Yaroch AL, Williams GC, Salkeld J, Toobert DJ, Domas A, Elliot DL, Hardin J, Nebeling L (2008). Accuracy and precision of two short screeners to assess change in fruit and vegetable consumption among diverse populations participating in health promotion intervention trials. J Nutr.

[B47] Linnan LA, Emmons KM, Klar N, Fava JL, LaForge RG, Abrams DB (2002). Challenges to improving the impact of worksite cancer prevention programs: comparing reach, enrollment, and attrition using active versus passive recruitment strategies. Ann Behav Med.

